# Mincocycline-Induced Discoloration of the Aorta

**DOI:** 10.1093/ofid/ofv129

**Published:** 2015-10-20

**Authors:** Suyog A. Mokashi, Taufiek Konrad Rajab, Peter S. Burrage, Annette K. Mizuguchi, Sari F. Aranki

**Affiliations:** 1 Division of Cardiac Surgery, Department of Surgery; 2 Departments of Anesthesiology; 3 Brigham and Women's Hospital and Harvard Medical School, Boston, Massachusetts

## CLINICAL VIGNETTE

**Figure 1.**
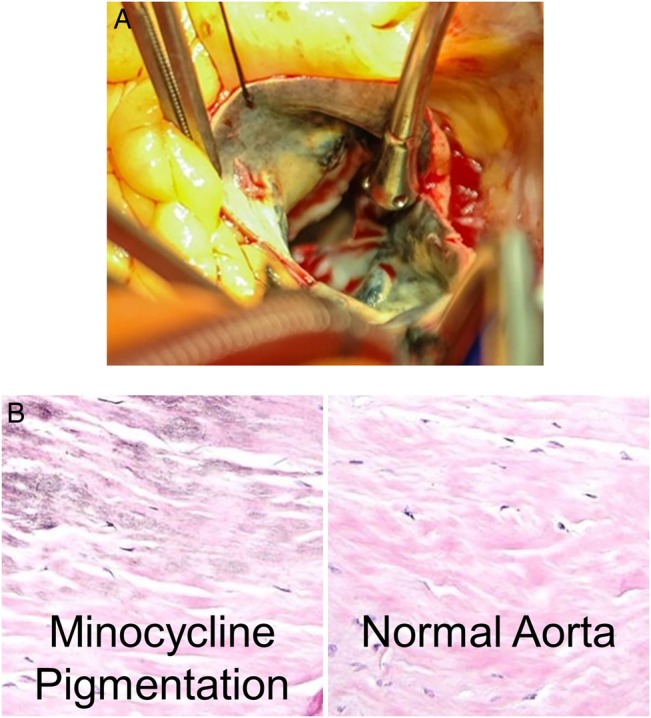
(A) Intraoperative photograph after aortotomy, showing slate-gray discoloration of the ascending aorta. (B) Histology shows the characteristic minocycline pigment associated with elastin and collagen as well as a section of normal aorta from the same patient.

A 64-year-old woman with known aortic insufficiency presented to our hospital with dyspnoea on exertion and chest pain. These symptoms had become progressively worse over several months. Her past medical history was otherwise notable for rheumatoid arthritis, treated with minocycline 100 mg PO BID and prednisone, as well as atrial fibrillation. She had started minocycline approximately 9 years prior to surgery and stopped it 6 months prior to surgery. Echocardiography showed severe aortic insufficiency and a decline in left ventricular ejection fraction from 60% 9 months prior to 50%. Coronary angiography was unremarkable apart from a 50% left anterior descending artery stenosis. She was referred for open aortic valve replacement and coronary artery bypass graft. During surgery, aortotomy revealed a slate-gray discoloration of the ascending aorta (Figure 1A). Further exploration showed that this discoloration did not involve the valve leaflets. The remainder of the procedure and her postoperative course were relatively unremarkable, and she was discharged on postoperative day 8. Pathological examination of the operative specimen confirmed minocyline-associated pigmentation (Figure 1B).

Minocycline is a broad-spectrum tetracycline antibiotic that is frequently used for the treatment of acne vulgaris and other infections. The drug is also recognized as a disease-modifying antirheumatic drug, and it is occasionally used for other inflammatory conditions. Prolonged use of minocycline can lead to slate-gray discoloration of skin and other tissues including the cardiac valves and coronary vessels [1, 2]. This is caused by deposition of degradation products of minocycline, which are chelated to iron and melanin. In this present article, we describe a case of minocycline-induced discoloration of the ascending aorta.

## CONSENT

The patient's written consent was obtained.
